# Early Postoperative PSA Dynamics and Prognostic Implications After Radical Prostatectomy

**DOI:** 10.3390/cancers18050850

**Published:** 2026-03-06

**Authors:** Yukun Tan, Qing H. Meng, Merve Dede, Ingold Huang, Hui Song, Ken Chen, Yu Zhang

**Affiliations:** 1 Department of Bioinformatics and Computational Biology, The University of Texas MD Anderson Cancer Center, Houston, TX 77030, USA; ytan1@mdanderson.org (Y.T.); mdede@mdanderson.org (M.D.); 2 Department of Laboratory Medicine, The University of Texas MD Anderson Cancer Center, Houston, TX 77030, USA; qhmeng@mdanderson.org; 3 Department of Pathology, Kaiser Permanente, Santa Clara, CA 95051, USA; ingold.x.huang@kp.org; 4 Department of Enterprise Data Engineering & Analytics, The University of Texas MD Anderson Cancer Center, Houston, TX 77030, USA; hsong@mdanderson.org

**Keywords:** prostate cancer, postoperative PSA dynamics, radical prostatectomy, clinical implications

## Abstract

**Background**: Serum PSA has been widely applied to monitor prostate cancer after treatment. Compared to a single static value, PSA dynamics may provide additional prognostic information. Although PSA velocity and doubling time have demonstrated prognostic value, their clinical utility is limited by the need for prolonged longitudinal PSA follow-up. This study aimed to evaluate the prognostic potential of early postoperative PSA dynamics following radical prostatectomy. **Methods**: A retrospective cohort study of 3474 patients who underwent radical prostatectomy with available pre- and postoperative serum PSA measurements was analyzed. A population-based approach was used to characterize early postoperative PSA dynamics and estimate the time required for PSA to decline to undetectable levels (<0.1 ng/mL). Accordingly, patients were classified into early remission (≤60 days), delayed remission (>60 days), or persistent PSA (failure to achieve undetectable PSA). Associations between postoperative PSA dynamics and clinical outcomes were evaluated. **Results**: Biochemical recurrence rates differed across the early remission, delayed remission, and persistent PSA groups (5.3%, 7.7%, and 24.0%). All-cause mortality similarly increased across these groups (1.1%, 1.9%, and 5.8%, respectively). Cox proportional hazards models confirmed the difference in recurrence-free and overall survival among PSA clearance groups. **Conclusions**: Early postoperative PSA dynamics after radical prostatectomy are strongly associated with recurrence and survival outcomes. Both PSA trajectory patterns and PSA clearance speed carry important prognostic information. Early postoperative PSA monitoring may support risk stratification and guide individualized postoperative surveillance.

## Simple Summary

Serum prostate-specific antigen (PSA) is widely used to monitor prostate cancer management. PSA dynamics, such as PSA velocity and doubling time, have demonstrated prognostic values. However, their clinical application is limited by the need for prolonged PSA follow-up. This retrospective study evaluated PSA trajectories and clearance speed immediately after radical prostatectomy and examined their association with clinical outcomes. With a population-based approach, the time required for PSA to become undetectable after surgery was estimated. Both PSA trajectory patterns and clearance speed were strongly associated with biochemical recurrence and survival outcomes. Early achievement of undetectable PSA was associated with more favorable outcomes, whereas delayed PSA remission or persistent PSA elevation was associated with an increased risk of recurrence and mortality. These findings highlight the prognostic value of early postoperative PSA dynamics, which may help with risk stratification and guide individualized postoperative surveillance.

## 1. Introduction

Prostate cancer is the most commonly diagnosed malignancy and the second leading cause of cancer-related death in men. In 2025, it was estimated that there were approximately 313,780 new cases and 35,770 deaths in the United States, accounting for nearly 30% of new cancer diagnoses and 11% of cancer-related deaths [1]. With early detection and effective treatment, the overall 5-year survival rate of prostate cancer has increased from 68% in 1975 to 97% in 2020 [1]. Despite these improvements, disease recurrence remains a common clinical challenge, with nearly 20–40% of patients experiencing relapse after initial local treatment, such as radical prostatectomy or radiotherapy [2,3,4,5]. Therefore, evaluation of treatment effectiveness and monitoring to detect potential relapses are critical components for prostate cancer management.

Active surveillance of prostate cancer currently relies on a combination of biopsy, imaging, and biomarkers [6,7]. Although biopsy remains the diagnostic gold standard, its invasive nature limits its suitability for frequent monitoring and introduces potential sampling bias. Imaging is less invasive but primarily detects macroscopic tumor burden at later stages. Biomarkers—primarily serum prostate-specific antigen (PSA) and related metrics such as PSA dynamics—provide a less invasive and more practical approach for longitudinal monitoring. PSA, a serine protease encoded by Kallikrein-related peptidase 3, is synthesized by epithelial cells of the prostate gland and secreted into seminal fluid under normal physiological conditions [8]. As prostate cancer disrupts the architecture of the prostate gland, including the basement membrane, PSA leaks into the circulation, resulting in elevated serum PSA levels. Consequently, serum PSA can be used to detect and monitor prostate cancer. In 1986, it was approved to monitor prostate cancer treatment by the Food and Drug Administration and has since been widely implemented in clinical practice [9]. However, PSA has its own limitations. While PSA is prostate-specific, it is not prostate cancer-specific, as benign conditions such as benign prostatic hyperplasia and prostatitis can also elevate PSA levels [10,11]. Furthermore, PSA levels vary among patients due to factors such as age, race, and physiological or pathological conditions. Therefore, rather than relying on a single PSA value, monitoring PSA dynamics may provide more informative insights for tumor monitoring.

Serial PSA measurements are routinely used to monitor disease progression, assess treatment response, and detect potential recurrence. Biochemical recurrence (BCR) is defined as a rising serum PSA level after definitive treatment, including radical prostatectomy, external beam radiation therapy, and brachytherapy, and often precedes clinically evident recurrence by several years [12,13,14]. Approximately 30–40% of patients with BCR will subsequently develop clinical recurrence within a median of 8 years [15,16]. The National Comprehensive Cancer Network (NCCN) guidelines recommend measuring serum PSA every 6–12 months for the first 5 years post-treatment and annually thereafter, or every 3 months for high-risk patients, to detect BCR [6]. Cumulative evidence has demonstrated that PSA velocity and doubling time are correlated with clinical outcomes [16,17,18]. However, their clinical applications remain limited due to their requirement of extended follow-up intervals [16,18,19]. To calculate PSA velocity or doubling time, it typically requires an additional 3–24 months of follow-up after PSA elevation, while clinical decisions often need to be made sooner, particularly in the early postoperative period. Prior studies have examined postoperative PSA levels following radical prostatectomy using predefined assessment windows, with time points ranging from approximately 6 weeks to 6 months after surgery [18,19,20,21,22]. While dichotomous classification of PSA status (detectable vs. undetectable) at selected postoperative time points has demonstrated clinical utility, characterization of early postoperative PSA dynamics may reveal additional clinically relevant insights.

This retrospective study aimed to evaluate postoperative PSA dynamics and determine whether early PSA clearance following surgery carries prognostic value and can provide timely insights for risk stratification and postoperative monitoring. A total of 3474 prostate cancer patients who underwent radical prostatectomy with PSA measurements obtained before and after surgery were included. Using a population-based approach, PSA was observed to decline to an undetectable level within 60 days for most patients. Based on clearance patterns, patients were classified into three groups: early remission (within 60 days), delayed remission (beyond 60 days), and persistent PSA. Associations between PSA clearance and clinical outcomes were subsequently assessed.

## 2. Materials and Methods

### 2.1. Study Design and Cohort Assembly

A retrospective cohort of men treated with radical prostatectomy for prostate cancer at MD Anderson Cancer Center between January 2018 and September 2025 was assembled. Radical prostatectomy was performed with curative intent. All data were extracted from the institution’s electronic health record system under institutional review board approval. All records, including demographic and clinical information, were deidentified prior to analysis.

Eligible patients were required to have a nonzero preoperative PSA value to exclude cases in which PSA was already undetectable for reasons unrelated to surgical resection. To ensure adequate postoperative assessment, patients were additionally required to meet at least one of the following criteria: (i) ≥1 undetectable postoperative PSA value (<0.1 ng/mL); (ii) ≥3 postoperative PSA measurements; or (iii) ≥1 postoperative PSA measurement obtained ≥200 days after surgery. These criteria were designed to exclude cases with insufficient postoperative follow-up to reliably characterize PSA responses.

### 2.2. PSA Measurement and Trajectory Pattern Definition

Serum PSA was measured using an electrochemiluminescence immunoassay on Roche Cobas analyzers (Roche Diagnostics, Indianapolis, IN, USA) with a laboratory-verified analytical measurement range from 0.1 to 90 ng/mL.

Postoperative PSA trajectories were classified as follows:

• Remission: PSA declined to an undetectable level (<0.1 ng/mL).
• Biochemical recurrence: Two consecutive PSA measurements demonstrating a rising pattern, with the first PSA ≥ 0.2 ng/mL and the second >0.2 ng/mL.
• Persistent PSA: Failure to achieve an undetectable PSA level despite adequate postoperative follow-up.
• Progression: Either a PSA value ≥ (dynamic nadir + 2.0 ng/mL) at any time after surgery or two consecutive PSA increases, each ≥ max (0.2 ng/mL, 20% of the preceding value).

### 2.3. Stepwise Population-Based Estimation and Categorization of PSA Clearance

For each patient, the earliest postoperative day with a recorded undetectable PSA value was identified. Patients who never achieved an undetectable PSA were followed through their last available postoperative PSA measurement.

To account for irregular timing and frequency of postoperative PSA testing across patients, time since surgery was partitioned into consecutive 10-day intervals. For each interval, the cumulative proportion of patients who had achieved an undetectable PSA by the end of that interval was estimated. The denominator at each time point included patients whose PSA status was known up to that interval, defined as those who (i) had at least one PSA measurement within the interval, (ii) had already achieved an undetectable PSA on or before the interval, or (iii) were documented to have persistently detectable PSA extending beyond the interval. The numerator included patients who had achieved an undetectable PSA on or before the end of the interval.

This approach yielded a stepwise, population-based estimate of the cumulative proportion of patients achieving undetectable PSA over time following radical prostatectomy, which indirectly reflects PSA clearance. Based on the observed inflection point in the population-level PSA clearance curve at approximately 60 days after surgery, patients were classified as early remission (within 60 days), delayed remission (beyond 60 days), or persistent PSA (failure to achieve undetectable PSA during the follow-up) for subsequent clinical outcome analysis.

### 2.4. Clinical Determination and Statistical Analysis

Clinical outcomes were primarily evaluated using recurrence-free survival (RFS) and overall survival (OS). RFS and OS were estimated with Kaplan–Meier methods and compared across groups. Associations between PSA clearance patterns and clinical outcomes were evaluated using Cox proportional hazards regression models, with age at treatment as a continuous covariate. Hazard ratios (HRs) with 95% confidence intervals (CIs) were reported. For descriptive comparisons, differences in recurrence and mortality proportions across PSA clearance subgroups were assessed using Pearson’s chi-squared tests with two-sided significance testing. A *p*-value < 0.05 was considered statistically significant.

The association between preoperative PSA level and time from surgery to first undetectable PSA was assessed using Spearman rank correlation analysis among patients who achieved an undetectable PSA within 500 days after surgery. Correlation coefficients (ρ) and two-sided *p*-values were reported.

## 3. Results

### 3.1. Study Cohort Assembly

**Figure 1:**
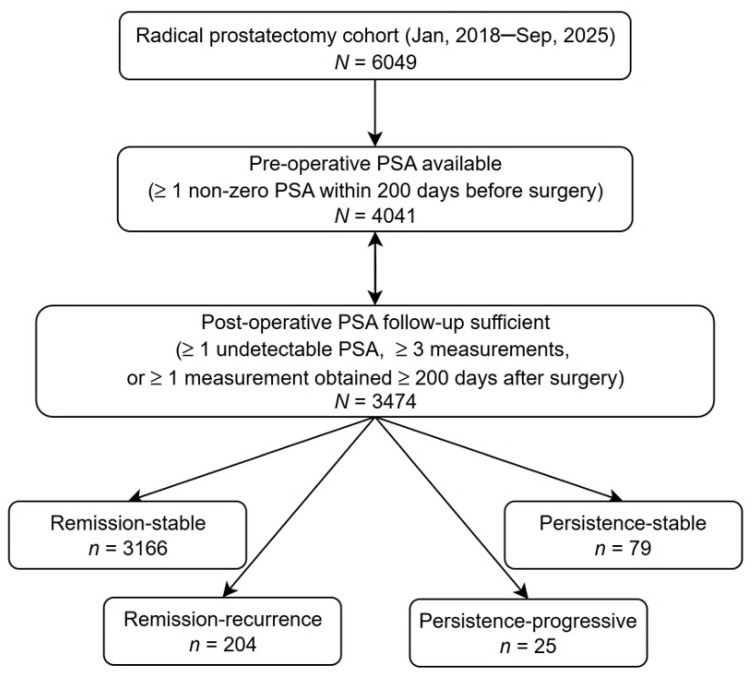
Study cohort flow diagram. From 6049 surgical cases to an analytic cohort of 3474 with adequate pre- and postoperative PSA measurements, then partitioned into remission-stable, remission-recurrence, persistence-stable, and persistence-progressive according to PSA trajectories.

Among 6049 men who underwent radical prostatectomy, 4041 met the preoperative PSA inclusion criteria, and 3474 satisfied postoperative follow-up adequacy requirements and were included in the analytic cohort (Figure 1). Age at surgery ranged from 37 to 82 years, with a median age of 63 years. This cohort included 2603 (74.9%) White, 517 (14.9%) Black, 126 (3.6%) Asian, and 228 (6.6%) patients of other or unknown races. Given its similarity to the racial distribution of the overall surgical prostatectomy population at the institution, the cohort was representative of the institution’s surgical prostatectomy population. The average follow-up duration was 1.9 years, with a maximum follow-up of 7.4 years.

### 3.2. Postoperative PSA Trajectories

Following radical prostatectomy, serum PSA levels declined markedly in most patients but demonstrated heterogeneous trajectories. 3370 patients (97.0%) achieved at least one undetectable postoperative PSA value (<0.1 ng/mL) and were classified as in remission, whereas 104 patients (3.0%) failed to reach an undetectable PSA level despite adequate postoperative follow-up (≥3 PSA measurements and/or ≥1 measurement obtained ≥ 200 days post-surgery) and were classified as having persistent PSA (Figure 1).

According to the American Urological Association (AUA) guidelines, biochemical recurrence after prostatectomy was defined as a rising PSA pattern on two consecutive measurements, with the first ≥0.2 ng/mL and the second >0.2 ng/mL; this definition also aligns with NCCN guidelines [6,13]. Among patients who achieved remission, 204 cases (5.9%) subsequently met criteria for biochemical recurrence, while 3166 (91.1%) remained biochemically stable throughout follow-up.

Among patients with persistent postoperative PSA elevation, 25 cases (0.7%) demonstrated further biochemical progression, defined by either (i) a PSA value ≥(dynamic nadir + 2.0 ng/mL) at any time after surgery or (ii) two consecutive PSA increases, each ≥ max (0.2 ng/mL, 20% of the preceding value). The remaining 79 cases (2.3%) with persistent PSA did not meet progressive criteria and were considered stable.

The proportion of patients experiencing biochemical recurrence among those who achieved remission was significantly lower than the proportion experiencing PSA progression among patients with persistent postoperative PSA (6.1% vs. 24.0%, *p* < 0.0001). Similarly, all-cause mortality was lower in the remission group than in the persistent PSA group (1.4% vs. 5.8%, *p* = 0.0003).

### 3.3. PSA Clearance and Clinical Outcomes

**Figure 2:**
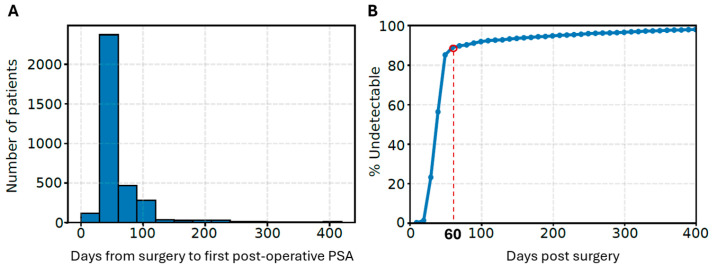
(**A**) The distribution of first postoperative PSA sampling. (**B**) Population-based PSA clearance curve. Each dot is the end of a 10-day bin, and the *y*-axis is the cumulative proportion of patients whose first undetectable PSA occurred on or before that day. The red circle highlighted the inflection point at 60 days after prostatectomy.

Next, postoperative PSA clearance and its association with clinical outcomes were examined. Owing to the retrospective nature of the study, postoperative PSA measurements were obtained at variable time points and frequencies across patients. With respect to the timing of the first postoperative PSA measurement, 3.4% of patients (*n* = 119) were tested within the first month after surgery, 68.3% (*n* = 2374) in the second month, 13.5% (*n* = 469) in the third month, and 14.7% (*n* = 512) beyond 3 months, including 2.0% (*n* = 71) tested more than 1 year after surgery (Figure 2A). As a result, it was not feasible to directly assess PSA clearance at the individual level.

To account for irregular postoperative sampling, we derived a stepwise, population-based estimate of the cumulative proportion of patients achieving undetectable PSA over time using 10-day time bins post radical prostatectomy (Figure 2B). The resulting stepwise cumulative curve demonstrated rapid PSA clearance during the early postoperative period. The median time to reach undetectable PSA was 48 days, and within approximately 60 days after surgery, most patients (88.6%) achieved undetectable PSA. Beyond 60 days, the slope of the PSA clearance curve flattened substantially, indicating slower conversion to undetectable PSA thereafter.

Based on this population-based PSA clearance pattern, patients were classified into three subgroups using the observed inflection point at 60 days after surgery as the cutoff for PSA clearance. The early-remission group included patients who achieved an undetectable PSA level within 60 days after radical prostatectomy (*n* = 2289; 65.9%); the delayed-remission group included those whose PSA reached an undetectable level after 60 days (*n* = 1081; 31.1%); and the persistent PSA group included patients who never reached an undetectable PSA level during follow-up (*n* = 104; 3.0%).

**Figure 3:**
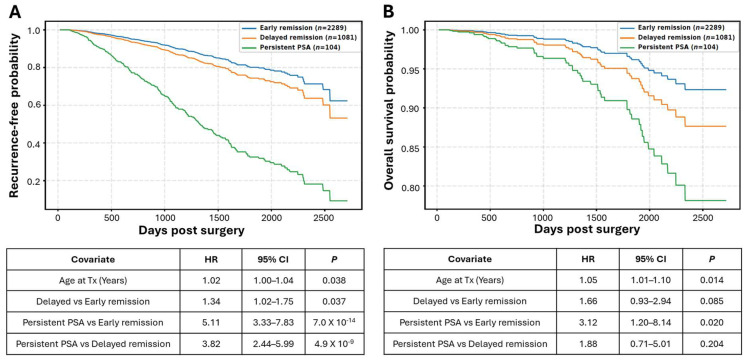
Kaplan–Meier analysis of recurrence-free survival (**A**) and overall survival probability (**B**) across the early-remission, delayed-remission, and PSA-persistent groups. Survival probability was plotted over time after radical prostatectomy. Recurrence event: biochemical recurrence for the early-remission and delayed-remission groups; progression for the persistent PSA group). HR, hazard ratio; CI, confidence interval; Tx, treatment/radical prostatectomy.

Across these groups, recurrence and overall survival demonstrated a graded pattern of risk. In terms of biochemical recurrence, 121 out of 2289 cases in the early-remission group experienced recurrence, which was significantly lower than in the delayed-remission group (5.3% vs. 7.7%, *p* = 0.0066). Both the early- and late-remission groups had substantially lower recurrence rates (5.3% and 7.7%, respectively) than the persistent PSA group (24.0%; both *p* < 0.0001). Although the crude recurrence rate differed modestly between the early- and delayed- remission groups, Kaplan–Meier analyses of recurrence-free survival demonstrated early separation of the curves that remained distinct over time (*p* < 0.05, Figure 3A). In Cox proportional hazards regression models, delayed remission was associated with a higher hazard of BCR compared with early remission (HR = 1.53; Figure 3A), indicating that even modest differences in early PSA clearance are associated with a higher underlying risk when evaluated on a time-to-event scale. Age, included as a continuous covariate in Cox models, showed a modest, linear association with recurrence risk, with each additional year at surgery corresponding to approximately a 2% increase in hazard (HR ≈ 1.02 per year).

Overall survival demonstrated a similar directional pattern (Table 1, Figure 3B). However, the number of deaths was low over the 2018 to 2015 follow-up period, limiting statistical power. Patients with persistent PSA had a significantly higher death rate compared with the early-remission group (5.8% vs. 1.1%; *p* = 0.0001) and lower overall survival over time (*p* = 0.020). Comparisons between the persistent PSA and delayed-remission groups, as well as between the delayed- and early-remission groups, exhibited higher mortality rates and lower overall survival, although these differences did not reach statistical significance.

Across analyses, early remission was consistently associated with the most favorable outcomes, delayed remission with intermediate risk, and persistent PSA with a substantially higher risk of recurrence and mortality.

### 3.4. PSA Clearance vs. Preoperative Burden

**Figure 4:**
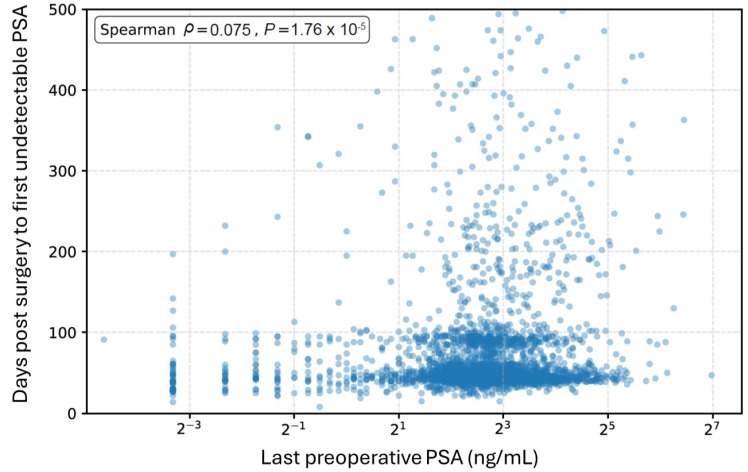
Association between preoperative PSA level and time to first undetectable PSA after radical prostatectomy. Scatterplot illustrating the relationship between the last preoperative PSA value (*x*-axis) and the time from surgery to first undetectable PSA (*y*-axis) among patients who achieved undetectable PSA within 500 days after surgery. A weak positive monotonic association was observed (Spearman correlation).

To evaluate whether slower postoperative PSA clearance simply reflected higher preoperative PSA burden, the association between the last preoperative PSA value and time from surgery to first undetectable PSA was evaluated among patients who achieved undetectable PSA within 500 days after radical prostatectomy (Figure 4). A Spearman correlation analysis demonstrated a weak positive association between preoperative PSA level and time to undetectable PSA (ρ = 0.075; *p* = 1.76 × 10^−5^). Although patients with higher preoperative PSA values tended to require a slightly longer time to reach an undetectable PSA, the magnitude of this association was small, indicating that baseline PSA burden accounted for only a limited proportion of the variability in postoperative PSA clearance time.

## 4. Discussion

This retrospective study analyzed 3474 patients who underwent radical prostatectomy with serum PSA monitored before and after surgery, revealing substantial heterogeneity in postoperative PSA trajectories and clearance dynamics. In the majority of patients (97.0%), serum PSA declined to an undetectable level following surgery, whereas 3.0% of patients failed to achieve undetectable PSA. Comparison between these two groups indicated that PSA persistence was associated with worse clinical outcomes, including disease progression and mortality. Within the remission group, PSA clearance occurred at variable rates. A stepwise, population-based estimate of the cumulative proportion of patients reaching undetectable PSA demonstrated that PSA clearance occurred within 60 days after surgery in most cases. Stratification into early remission (within 60 days), delayed remission (beyond 60 days), and persistent PSA groups revealed that rapid PSA clearance was associated with better clinical outcomes, including a low rate of recurrence and mortality.

These findings indicate that beyond static PSA levels, PSA dynamics—particularly PSA clearance speed—carry important prognostic information. Although PSA persistence identifies a high-risk subgroup, this group represents only a small fraction of patients (approximately 3.0%), leaving the majority of post-prostatectomy patients insufficiently stratified by this binary classification alone. Moreover, PSA persistence can only be determined after months of follow-up, limiting its utility for early risk assessment and clinical decision-making. In contrast, PSA dynamics enable additional risk stratification within the remission population. Unlike prior studies that focused primarily on PSA recurrence metrics, such as PSA velocity or doubling time, this study specifically examined PSA clearance immediately after prostatectomy and demonstrated that clearance speed carries prognostic value. Compared with recurrence-based metrics, PSA clearance reflects early postoperative tumor burden and surgical effectiveness, thereby providing more timely insights for risk stratification and postoperative clinical surveillance. The data suggests that closer PSA monitoring within the first three months after surgery can be clinically meaningful and that surveillance frequency and intervals could be tailored according to PSA clearance patterns.

To address the challenges of sparse, irregular, and non-uniform PSA testing inherent to retrospective studies, we developed a stepwise, population-based method to estimate the cumulative proportion of patients achieving an undetectable PSA. This approach reflects the population-level dynamics of PSA clearance indirectly and enables an objective definition of early and delayed remission. Notably, preoperative PSA levels showed only a weak association with time to PSA clearance, indicating that factors beyond PSA burden—such as tumor biology, pathological features, and surgical completeness—are likely contributors. The comparison between early and delayed remission supports the interpretation that early achievement of undetectable PSA, particularly within 60 days, more accurately reflects surgical effectiveness and provides prognostic information beyond baseline PSA levels alone.

Multiple prognostic factors have been established to correlate with clinical outcomes following radical prostatectomy [4,16,17,18,19,23,24,25,26,27]. Some of them reflect tumor features, such as tumor stage, grade, and Gleason score, whereas postoperative features reflect surgical and pathological findings, such as margin status, seminal vesicle invasion, and lymph node involvement [24,25,26,27,28,29]. In addition, both preoperative PSA level and BCR-based PSA dynamics (PSA velocity and PSA doubling time) have demonstrated prognostic relevance [16,17,18,20,30,31]. This study suggests that early postoperative dynamics may represent another prognostic dimension reflecting early treatment response in a minimally invasive manner. However, it remains to be determined whether it could provide additional values beyond established prognostic factors by either enabling further risk stratification or reflecting combined clinicopathologic features. In this retrospective study, certain clinicopathologic variables, including detailed pathologic staging and grading parameters, were not uniformly available across the study period, limiting formal multivariable adjustment and integrated prognostic modeling. Future investigations incorporating more comprehensive datasets and multivariable modeling approaches would help clarify the relationship between early postoperative PSA dynamics and established prognostic factors.

Several limitations should be acknowledged. This was a single-center retrospective study, and despite the relatively large sample size, the findings may not be fully generalizable to other patient populations. Variations in patient demographic characteristics (including age, race/ethnicity, and genetic ancestry), tumor biology (such as grade and stage), and surgical management across institutions may influence PSA clearance patterns or kinetics, which warrants further investigation. In addition, the limited follow-up duration or missing information restricted the evaluation of long-term clinical outcomes, including cancer-specific survival. As an observational retrospective analysis, the results may also be influenced by residual confounding from unmeasured clinical variables, such as variations in postoperative management and adjuvant therapies. Although cohort assembling criteria were designed to improve the interpretability of PSA dynamics, the potential effects of such factors cannot be fully excluded. Future studies incorporating extended follow-up and more comprehensive clinical datasets would further strengthen understanding of the long-term prognostic implications of early postoperative PSA dynamics.

PSA varies substantially among individuals and is influenced by diverse physiological and pathological conditions. Therefore, rather than relying solely on static PSA levels or fixed thresholds, assessment of PSA dynamics may provide greater diagnostic and prognostic value. With advances in artificial intelligence and longitudinal data modeling, individualized PSA dynamics profiling may become both technically feasible and clinically impactful.

## 5. Conclusions

Serum PSA plays an irreplaceable role in prostate cancer monitoring. Beyond absolute one-time PSA levels, this study demonstrates PSA dynamics—particularly postoperative clearance speed—carry significant prognostic value. Close monitoring of PSA immediately after radical prostatectomy may not only improve risk stratification but also inform more personalized surveillance strategies for patients with prostate cancer.

## Figures and Tables

**Table 1:** Clinical outcomes across postoperative PSA dynamics.

Group	Patients (*n*)	Recurrence (*n*)	Recurrence (%)	Deaths (*n*)	All-Cause Mortality (%)
Early remission	2289	121	5.3%	26	1.1%
Delayed remission	1081	83	7.7%	21	1.9%
Persistent PSA	104	25	24.0%	6	5.8%

## Data Availability

The raw data supporting the conclusions of this article will be made available by the authors on request.

## Abbreviations

The following abbreviations are used in this manuscript:

PSA	Prostate-specific antigen
NCCN	National Comprehensive Cancer Network
ASTRO	American Society of Radiation Oncology
AUA	American Urological Association
RP	Radical prostatectomy
RT	Radiation therapy
BCR	Biochemical recurrence
RFS	Recurrence-free survival
OS	Overall survival
HR	Hazard ratio
CI	Confidential interval
